# Postmortem cardiac imaging in fetuses and children

**DOI:** 10.1007/s00247-014-3164-0

**Published:** 2015-04-01

**Authors:** Andrew M. Taylor, Owen J. Arthurs, Neil J. Sebire

**Affiliations:** 1 Cardiorespiratory Division, Level 7, Old Nurses Home, Great Ormond Street Hospital for Children NHS Foundation Trust, London, UK; 2 UCL Institute of Cardiovascular Science, London, UK; 3 Department of Radiology, Great Ormond Street Hospital for Children NHS Foundation Trust, Great Ormond Street, London, WC1N 3JH UK; 4 Department of Histopathology, Great Ormond Street Hospital for Children NHS Foundation Trust, London, UK

**Keywords:** Postmortem imaging, Magnetic resonance imaging, Computed tomography, Autopsy, Pathology, Fetus, Child

## Abstract

Fetal and pediatric cardiac autopsies have a crucial role in the counseling of parents with regard to both the cause of death of their child and the implications of such findings for future pregnancies, as well as for quality assurance of antenatal screening programs and antemortem diagnostic procedures. Postmortem imaging allows an opportunity to investigate the heart in situ prior to dissection, and both postmortem CT and postmortem MRI have shown excellent accuracy in detecting the majority of clinically significant cardiac lesions in the perinatal and pediatric population. As less-invasive autopsy becomes increasingly popular, clinical guidelines for maximal diagnostic yield in specific circumstances can be developed.

## Introduction

Fetal and pediatric cardiac autopsies have a crucial role in the counseling of parents with regard to both the cause of death of their fetus or child and the implications of such findings for future pregnancies [1, 2], as well as for quality assurance of antenatal screening programs and antemortem diagnostic procedures. Cardiac abnormalities are found in up to 35% of fetal autopsies [3, 4], and only about 50% of those abnormalities can be detected antenatally [5, 6]. Furthermore, cardiac defects are seen at autopsy in approximately 10% of sudden deaths in infants [6, 7] and could be the cause of death in up to 84% of these cases [8]. Even though the vast majority of such abnormalities are structural, only 40% are detected before death [7]. There is growing interest in using postmortem imaging as an alternative for conventional autopsy [9], in parallel to the significant reduction in fetal and pediatric autopsy update in recent years, and here we review the recent publications regarding cardiac postmortem imaging in children.

## Previous studies and limitations

Several authors have historically reported poor accuracy of postmortem cardiovascular MRI imaging. Most published data on cardiac postmortem MRI have been limited to conventional 2-D sequences and show extremely poor sensitivity (around 12%) for the detection of structural cardiac abnormalities [10]. For example, Alderliesten et al. [11] compared cardiac postmortem MRI with conventional autopsy in 26 fetuses, with 5 cardiac abnormalities identified at autopsy but none detected by cardiac postmortem MRI — sensitivity 0%. Breeze et al. [12] compared whole-body postmortem MRI and autopsy in 36 fetuses. Of the 8 fetuses who had cardiac lesions, only 2 were detected by postmortem MRI — 25% sensitivity. Cohen et al. [13] reported 2 cases (no cardiac lesions) of cardiac postmortem MRI in sudden infant death in which both cases were non-diagnostic. All of these studies used 2-D cardiac postmortem MRI with low resolution (slice thickness 2–5 mm), with images reported by general radiologists. It is likely that more-detailed imaging, with 3-D reconstruction and interpreted by dedicated cardiac imaging specialists, would yield higher diagnostic accuracy. Three-dimensional instead of 2-D imaging should enable accurate identification of complex structures in any imaging plane. In postmortem MRI, higher resolution and longer scan times can be used so that partial volume effects are minimized, enhancing the potential to accurately identify small structures.

## 3-D cardiac postmortem MRI

MARIAS (magnetic resonance imaging autopsy study) compared the diagnostic accuracy of 3-D cardiac postmortem MRI with conventional autopsy and histopathology assessment in fetuses and children [14]. An isotropic 3-D heavily T2-weighted gradient-echo sequence was employed to assess the heart in detail, with imaging times of 30 min or more needed to generate the combination of high signal and very high spatial resolution, although exact parameters will vary between machines and manufacturers. In our study we used a 3-D constructive interference in steady state (CISS) sequence (two steady-state free precession sequences acquired with different radio frequency pulses and then combined for heavily T2-weighted 3-D images) to acquire 0.6-mm isotropic voxels in about 25 min using a conventional 8-channel phased-array coil; the study protocols have been published [15].

**Fig. 1:**
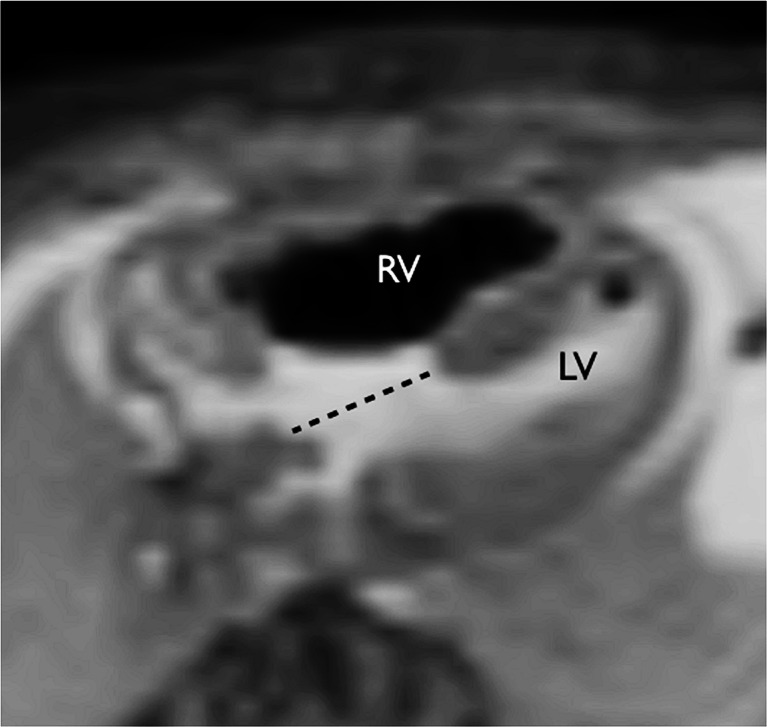
Complete atrioventricular septal defect (*dotted line*) on axial T2-weighted postmortem MRI in a 22-week gestation fetus with trisomy 21. Note air in the right ventricle (*RV*) secondary to fetocide injection and a trace of pericardial effusion. *LV* left ventricle. Reproduced with permission [14]

**Fig. 2:**
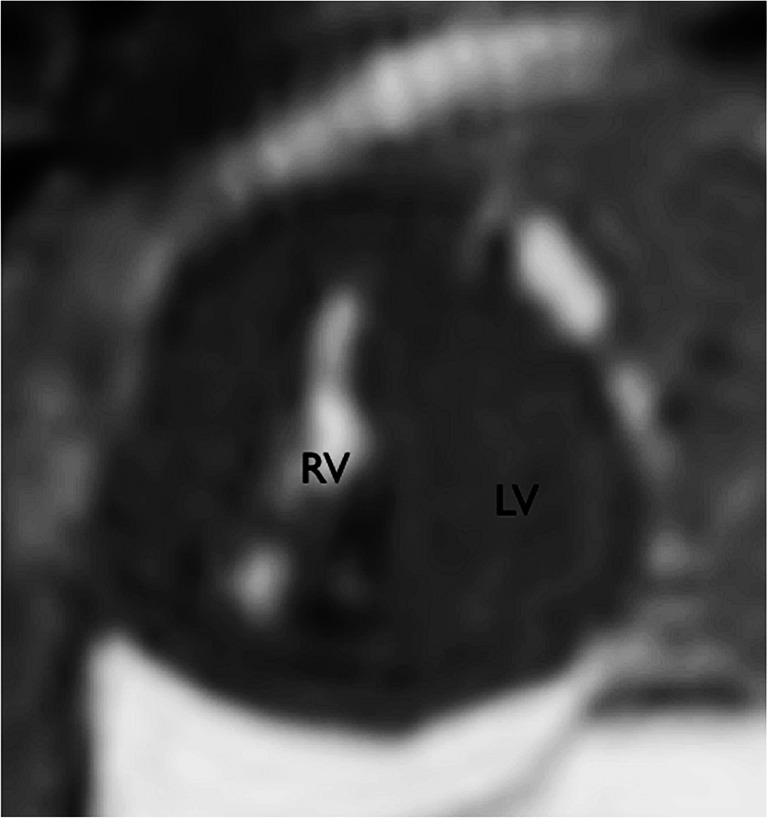
Fetal cardiac postmortem MRI shows hypoplastic left heart syndrome on sagittal T2-weighted image in a 22-week gestation fetus. *LV* left ventricle, *RV* right ventricle. Reproduced with permission [14]

**Fig. 3:**
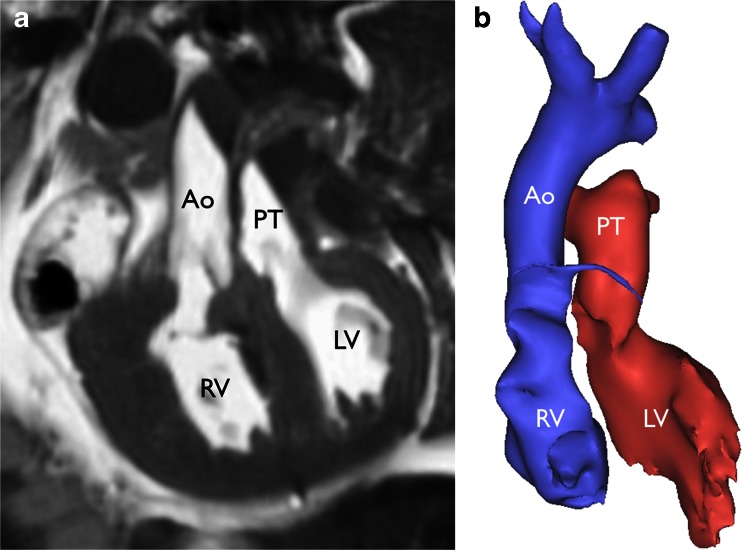
Neonatal cardiac postmortem MRI in a 1-day-old neonate with transposition of the great arteries. Coronal T2-weighted image (**a**) and 3-D volume-rendered reconstruction (**b**) show the aorta (*Ao*) arising from the right ventricle (*RV*) and the pulmonary trunk (*PT*) arising from the left ventricle (*LV*). Reproduced with permission [14]

**Fig. 4:**
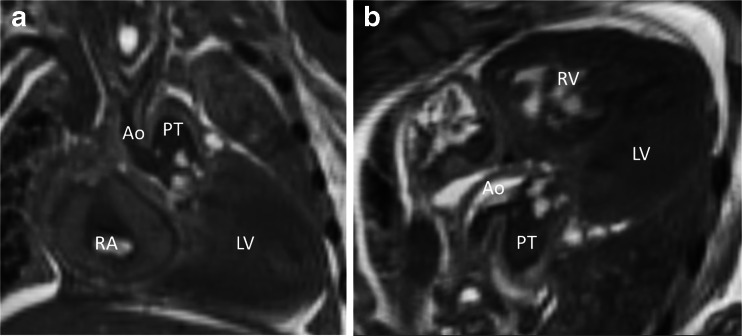
Neonatal cardiac postmortem MRI in a 10-day-old neonate who died unexpectedly at home. Coronal (**a**) and oblique axial (**b**) T2-weighted images show truncus arteriosus. The head and neck arterial vessels (*Ao*) arise anteriorly from the common trunk (*Ao/PT*) and the descending aorta posteriorly from the pulmonary arterial trunk (not shown). *LV* left ventricle*, RA* right atrium, *RV* right ventricle

**Fig. 5:**
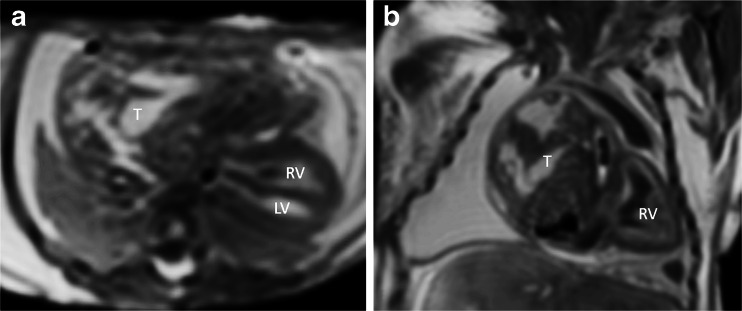
Fetal cardiac postmortem MRI in a 29-week gestation fetus shows a large cardiac teratoma (*T*) following unsuccessful in utero laser ablation. The large teratoma displaces the heart posteriorly and laterally on (**a**) axial and (**b**) oblique coronal T2-weighted images. *LV* left ventricle*, RV* right ventricle

**Fig. 6:**
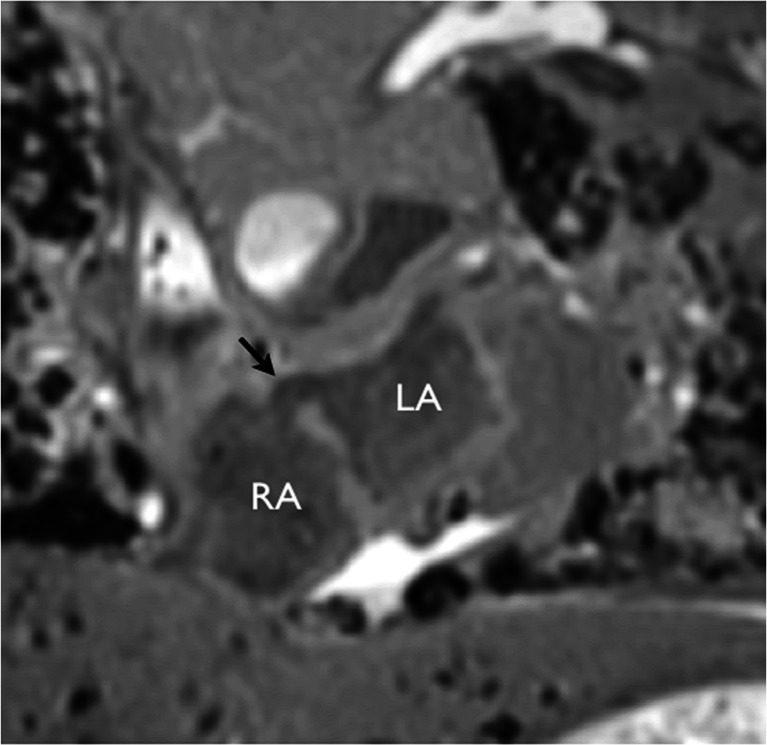
Potential overcall by cardiac postmortem MRI. A high atrial septal defect (*arrow*) was called on coronal T2-weighted postmortem MRI. Conventional autopsy of the heart was reported as normal. *LA* left atrium, *RA* right atrium

**Fig. 7:**
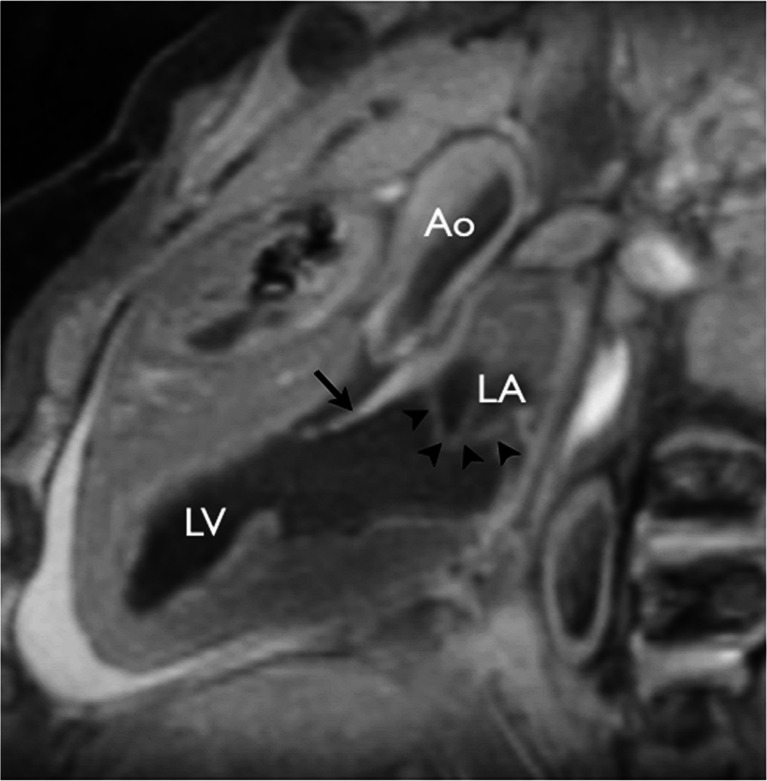
Potential overcall by cardiac postmortem MRI of cor triatriatum (*left* atrial membrane, *arrowheads*) was reported on oblique sagittal T2-weighted imaging. Long-axis view shows the *left* ventricle (*LV*), left atrium (*LA*) and aorta (*Ao*). *Arrow* indicates the open anterior leaflet of the mitral valve. Conventional autopsy of the heart was reported as normal. Reproduced with permission [14]

Contrary to previous reports, in this large study cardiac postmortem MRI was shown to have good diagnostic utility (Figs. 1, 2, 3, 4, 5, 6 and 7). On detailed analysis of 400 cases, cardiac postmortem MRI failed to detect only 2 cases of significant structural heart disease abnormalities (both tetralogy of Fallot; false negatives), and both of these were in small fetuses ≤24 weeks of gestation. One minor cardiac abnormality was missed (a ventricular septal defect). There were 13 overcalls (false positives) of structural heart disease, of which only 1 was for complex congenital heart disease (cor triatriatum; Fig. 7). The other overcalls were for minor congenital heart defects including ventricular septal defect, atrial septal defect (Fig. 6), coarctation, partial anomalous venous drainage and aortic stenosis. All but one overcall occurred in fetuses [16].

Several cardiac postmortem MRI features were considered to be postmortem artefact and are probably better visualized on cardiac postmortem MRI than at conventional autopsy. These included small pericardial and pleural effusions, intracardiac air, and blood clots. Similarly, both ventricular walls may have a thickened appearance after death and were interpreted as normal postmortem change rather than ventricular hypertrophy. Intracardiac air is also often seen as a normal postmortem finding, although the precise origin of the air or gas, and its clinical significance, remain unclear. Without extensive putrefaction, there is usually insufficient volume of gas for it to generate sufficient contrast as a substitute for an intravascular contrast agent, although it may be more apparent on postmortem CT than postmortem MRI.

Crucially, in the MARIAS study a normal scan in fetuses >24 weeks’ gestation, newborns and children meant that there was no structural cardiac abnormality. However, while cardiac postmortem MRI yielded excellent results for structural heart disease, conventional autopsy identified myocarditis as the cause of mortality in 2% of children (neonates >1 year of age to ≤16 years of age) and no myocarditis was detected at cardiac postmortem MRI [16]. More advanced imaging techniques are likely to be required to identify myocardial wall abnormalities, including myocarditis and ischemia. We would argue that the good diagnostic utility seen in this study is largely attributable to the use of high-resolution, detailed imaging with 3-D reconstruction reported by a dedicated cardiac postmortem MRI specialist.

## Use of postmortem CT angiography

**Fig. 8:**
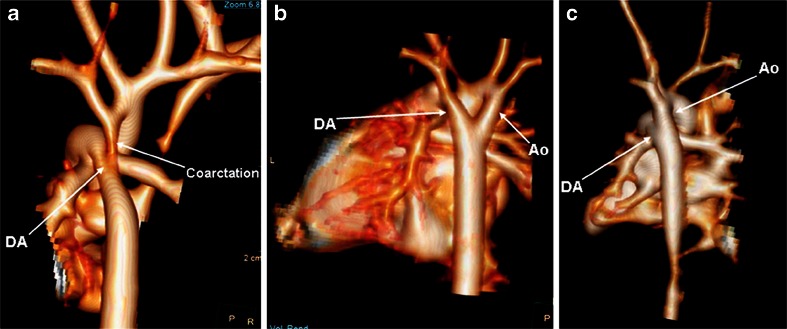
Fetal postmortem CT angiography of arch abnormalities. Postmortem CT angiography with 3-D visualization shows (**a**) coarctation of the aorta in a fetus at 24 weeks’ gestation, (**b**) right aortic arch in a fetus at 25 weeks’ gestation, and (**c**) normal great vessels in a fetus at 25 weeks’ gestation. *Ao* aorta, *DA* ductus arteriosus. Reproduced with permission [17]

Contrast-enhanced postmortem CT angiography is used primarily to visualize cardiac structures in adults. However, the delivery of contrast medium can be challenging in small fetuses and neonates. Postmortem CT angiography is becoming the gold standard for adult postmortem imaging, with intravenous contrast agent administered via femoral access [17, 18] or a large neck vessel. In fetuses and neonates, contrast agent can be injected via the umbilical vessels, although in late-gestation fetuses direct intracardiac contrast injection may be preferred (Fig. 8) [19, 20].

Cardiac anatomy including heart situs, the four-chamber view and great vessels was visualized on postmortem CT angiography in 29 of 33 fetuses (87.9%) in one study using a direct intracardiac injection technique, with congenital cardiac anomalies identified in all cases [19]. The first uses of whole-body perinatal postmortem CT angiography are now being described [21], although a diagnostic accuracy of postmortem CT angiography versus postmortem MRI for congenital cardiac disease remains to be studied. The ultimate choice of perinatal postmortem CT angiography or postmortem MRI will largely depend on operator experience, but postmortem MRI may be preferred because is it less invasive.

## Potential limitations of postmortem cardiac imaging

Although cardiac postmortem MRI has good diagnostic accuracy for congenital heart disease, there are several limitations to this technique.

The first issue relates to fetal size. Ten percent of total cardiac postmortem MRI scans were non-diagnostic in the MARIAS study, and all related to smaller fetuses <24 weeks’ gestation. In addition, the 3 structural heart disease misses and 5 of the 13 overcalls (38%) were made in this group [16]. Poor image resolution was thought to account for these errors, although most cases were still diagnostic. The underlying problem of image resolution and size in this group could be addressed by developing postmortem CT angiography, or carrying out postmortem MRI at high field strength [22], which is discussed later in this manuscript. High field postmortem MRI has particular promise in very small fetuses and in the investigation of complex cardiac abnormalities.

The second issue relates to false-positive results. Outside of fetuses ≤24 weeks’ gestation, only one major structural abnormality was overcalled (cor triatriatum) with the remaining overcalls being septal defects (atrial or ventricular), one coarctation, aortic stenosis and one case of partial anomalous pulmonary venous drainage [16]. All of these false-positive cardiac MRI diagnoses could result from inadequate image resolution, over-interpretation by the reporter, or a miss of a true cardiac lesion at conventional autopsy. All the lesions identified, including cor triatriatum (a rare diagnosis with a flimsy membrane in the left atrium that could be missed or destroyed during the conventional autopsy), could easily have been missed at conventional autopsy (Fig. 7). In particular, the diagnosis of coarctation is difficult and subjective at both cardiac MRI and conventional fetal autopsy.

The third issue relates to diagnoses that were consistently missed by cardiac postmortem MRI and for which tissue histology is always likely to be needed. In particular, myocarditis was consistently missed in 8 out of 90 infants and children (almost 10% of childhood cases in this study; 2% of total cases [16]). At present, cardiac tissue examination is necessary in all children, should a diagnosis of structural heart disease not be made (myocarditis was not seen in any older case with structural heart disease). This may be possible using a less-invasive laparoscopic approach [23], or in selected cases percutaneous biopsy under image guidance may be a satisfactory alternative to open dissection and biopsy [24], but this requires further proof of principle. Developments in tissue characterization of myocarditis and myocardial damage in the living using T1 and T2 mapping and magnetization transfer MRI methods [25] could allow identification of myocarditis by postmortem MRI to become possible, but it is not yet available.

## Advances in postmortem cardiac imaging

Several areas of imaging show promise for investigation of very small fetuses, particularly MRI at higher field strength than 1.5 T. Cardiac postmortem MRI of the fetal heart at 3 T has been documented in two recent studies from the same group in Leuven, Belgium. They showed in a study of 39 fetuses the feasibility of performing accurate measurements on all major structures in fetuses beyond 14 weeks [26]. They also showed, in a study of 24 fetuses, the feasibility of diagnosing complex congenital heart disease in fetuses as young as 16 weeks’ gestation [27]. However, cardiac postmortem MRI was non-diagnostic in three cases of valve pathology in early gestation fetuses.

One of the main limitations of postmortem MRI at both 1.5 T and 3.0 T is adequate evaluation of fetal cardiac anatomy: only the heart situs and four-chamber view can be consistently visualized fewer than 16 weeks’ gestation. In contrast, using high-field postmortem MRI at 9.4 T, the four-chamber view and outflow tracts can be visualized in all fetuses irrespective of gestational age [28]. By directly comparing cases at 1.5 T and 9.4 T, in a group of fetuses <22 weeks’ gestation, conventional postmortem MRI at 1.5 T was non-diagnostic in 14 out of 18 cases that were all diagnostic at 9.4 T and at autopsy [22]. High-field postmortem MRI at 9.4 T may therefore be an acceptable alternative to invasive autopsy for fetuses with congenital heart disease at or below 20 weeks’ gestation [22].

Micro-CT is another potential alternative diagnostic modality for imaging small objects, using CT, but at improved resolution down to micrometers rather than millimeters [29]. Developed for small animal imaging, higher spatial resolution than conventional CT now allows detailed isotropic anatomical images of the heart, and contrast-enhanced micro-CT imaging in neonatal mice has been shown to reliably detect a wide spectrum of congenital heart disease. The largest animal study of congenital heart disease was recently published by Kim et al. [30], where 307 out of 2,105 fetal and newborn mice were diagnosed with ventricular septal defects with an overall accuracy of 89.8%; there was a 97.4% accuracy for identifying double outlet right ventricle (*n* = 36), transposition of the great arteries (*n* = 14), and persistent truncus arteriosus (*n* = 3) and a 99.6% accuracy for diagnosing arch abnormalities.

**Fig. 9:**
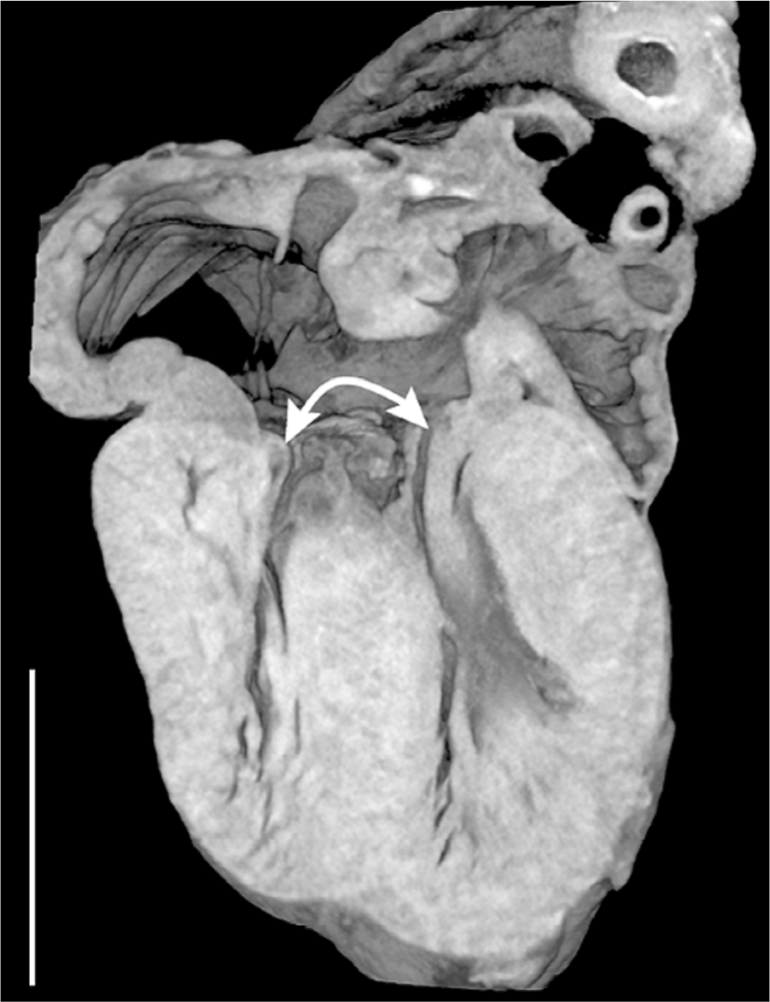
Micro-CT of a human fetal heart at 16 weeks’ gestation shows atrioventricular septal defect with a common atrioventricular valve (*curved arrow*). Scale bar = 1 cm. Reproduced with permission [28]

Micro-CT of human fetal hearts has now been shown to be possible [31] (Fig. 9), although extracting and fixing tissue for optimal contrast is necessary. The utility of postmortem micro-CT compared to high-field postmortem MRI will be an interesting area of diagnostic study in the near future.

Although extensive use is made of sonography of the heart (echocardiography, or echosonography) both antenatally and in pediatric patients, and it would be logical to apply similar techniques in the postmortem setting, there is no literature on the role of postmortem cardiac sonography. In experienced hands, postmortem echocardiography could have a similar diagnostic yield in death as in life, although this may be limited to anatomical defects rather than physiological abnormalities.

## Clinical utility and implications

Even without the use of high field MRI, cardiac postmortem MRI could be used as the first-line assessment for structural heart disease for all fetuses and neonates. If all non-diagnostic and positive cardiac postmortem MRI scans were referred for conventional autopsy, very few diagnoses would be missed from the current evidence available. If 9.4-T imaging would reduce the number of non-diagnostic cardiac postmortem MRIs and provide diagnostic accuracy similar to that found in older fetuses and neonates, and given that some of the overcalls are conventional autopsy misses, then cardiac postmortem MRI could be seen as a replacement for conventional autopsy.

Cardiac anomalies are often present in fetuses with extracardiac malformations, and this can be missed or wrongly interpreted on antenatal scans [4, 32], thus cardiac postmortem MRI is crucial to find a correct diagnosis in such cases and becomes acceptable to most parents [33–35]. Additional findings from cardiac postmortem MRI scans can change the antemortem diagnosis and therefore even alter the recurrence risks for future pregnancies [4].

Conventional fetal and pediatric autopsies are performed only in specialized centers and often require transfer of bodies over long distances and consequent delay in the funeral. Unlike antemortem cardiac MRI, postmortem MRI can be performed in local hospitals with minimal training of radiographers and images transferred to the specialist pediatric cardiac postmortem MR imager for interpretation. Total time for cardiac postmortem MRI may be more than an hour; nevertheless postmortem MRI is often performed out of hours and little monitoring is needed during the scan, so scan time may be of little significance.

Ultimately a step-wise process to the less-invasive autopsy should be considered, perhaps starting with postmortem sonography or CT. In cases where cardiac disease is suspected, dedicated cardiac postmortem MRI or postmortem CT angiography should be performed, with recognition of their limitations, and then less-invasive sampling of the heart should occur, with progression to a full conventional autopsy if needed. This step-wise approach is likely to garner parental approval [33]. Furthermore, the use of cardiac postmortem MRI as a routine adjuvant to autopsy may improve the accuracy of cardiac autopsy by guiding pathologists to specific pathological lesions. By providing a permanent, reproducible data store with reconstruction in any plane from a 3-D acquisition, cardiac postmortem MRI in fetuses and children can offer a great deal of diagnostic information to the current perinatal autopsy.

## Conclusion

Three-dimensional cardiac postmortem MRI can provide equivalent structural information to that of conventional autopsy in the majority of larger fetuses, newborns and children. This technique may have a major role in developing less-invasive autopsy methods. Moreover, routine use of cardiac postmortem MRI as an adjuvant to conventional autopsy may increase the yield from conventional autopsy. Further study of high-field postmortem MRI, postmortem CT and micro-CT will continue to optimize the best methods for this form of less-invasive postmortem assessment.
